# The *ABCA1* Gene R230C Variant Is Associated with Decreased Risk of Premature Coronary Artery Disease: The Genetics of Atherosclerotic Disease (GEA) Study

**DOI:** 10.1371/journal.pone.0049285

**Published:** 2012-11-09

**Authors:** Teresa Villarreal-Molina, Carlos Posadas-Romero, Sandra Romero-Hidalgo, Erika Antúnez-Argüelles, Araceli Bautista-Grande, Gilberto Vargas-Alarcón, Eric Kimura-Hayama, Samuel Canizales-Quinteros, Juan Gabriel Juárez-Rojas, Rosalinda Posadas-Sánchez, Guillermo Cardoso-Saldaña, Aída Medina-Urrutia, María del Carmen González-Salazar, Rocío Martínez-Alvarado, Esteban Jorge-Galarza, Alessandra Carnevale

**Affiliations:** 1 Laboratorio de Genómica de Enfermedades Cardiovasculares, Instituto Nacional de Medicina Genómica (INMEGEN), Mexico City, Mexico; 2 Departmento de Endocrinología, Instituto Nacional de Cardiología “Ignacio Chávez” (INCICH), Mexico City, Mexico; 3 Departmento de Genómica Computacional, INMEGEN, Mexico City, Mexico; 4 Departamento de Biología Molecular, INCICH, Mexico City, Mexico; 5 Departmento de Tomografía Cardíaca, INCICH, Mexico City, Mexico; 6 Departamento de Biología, Facultad de Química, Universidad Nacional Autónoma de México (UNAM), Mexico City, Mexico; 7 Unidad de Biología Molecular y Medicina Genómica, Instituto Nacional de Ciencias Médicas y Nutrición “Salvador Zubirán”, Mexico City, Mexico; 8 Dirección de Investigación, INMEGEN, Mexico City, Mexico; Leibniz-Institute for Arteriosclerosis Research at the University Muenster, Germany

## Abstract

**Background:**

*ABCA1* genetic variation is known to play a role in HDL-C levels and various studies have also implicated *ABCA1* variation in cardiovascular risk. The functional *ABCA1*/R230C variant is frequent in the Mexican population and has been consistently associated with low HDL-C concentrations. Although it has been associated with other cardiovascular risk factors such as obesity and type 2 diabetes mellitus, it is not known whether it is associated with coronary artery disease (CAD).

**Aim:**

The purpose of the study was to analyze whether the *ABCA1*/R230C variant is associated with premature CAD in a case-control association study (GEA or Genetics of Atherosclerotic Disease), and to explore whether BMI modulates the effect of the C230 allele on other metabolic traits using a population-based design.

**Results:**

The C230 allele was significantly associated with both lower HDL-C levels and a lower risk of premature CAD as compared to controls (OR = 0.566; *P_add_* = 1.499×10^−5^). In addition, BMI modulated the effect of R230C on body fat distribution, as the correlation between BMI and visceral to subcutaneous adipose tissue (a metric of the propensity to store fat viscerally as compared to subcutaneously) was negative in RR homozygous individuals, but positive in premenopausal women bearing the C230 allele, with a statistically significant interaction (*P* = 0.005). BMI-R230C interaction was also significant for triglyceride levels in women regardless of their menopausal status (*P = *0.036).

**Conclusion:**

This is the first study assessing the effect of the R230C/*ABCA1* variant in remature CAD. C230 was associated with both decreased HDL-C levels and a lower risk of premature CAD, and gender-specific BMI-R230C interactions were observed for different metabolic traits. These interactions may help explain inconsistencies in associations, and underscore the need to further analyze interactions of this functional and frequent variant with diet, exercise and other environmental factors.

## Introduction

Epidemiological studies consistently demonstrate that a low plasma level of high density lipoprotein-cholesterol (HDL-C) is associated with increased risk of ischemic heart disease (IHD) [1]. Although various studies have shown associations of diverse genetic variants with plasma HDL-C levels, they do not always change the risk of myocardial infarction [2]. The ATP-binding cassette A1 transporter (*ABCA1*) is a highly polymorphic trans-membrane protein that mediates the cellular efflux of cholesterol and phospholipids to lipid poor HDL apolipoproteins [3]. Both rare and common *ABCA1* genetic variation play a role in HDL-C levels in the general population [4], [5]; and various studies have implicated *ABCA1* gene variation in cardiovascular risk [5]–[12].

The R230C variant of the *ABCA1* gene is of particular interest in the Americas because it is private to Native American and descendant populations and is frequent in the Mexican-Mestizo population (∼10%). In addition, the 230C allele has a functional effect decreasing cholesterol efflux by approximately 30% *in vitro* and shows evidence of positive selection in Native Americans [13]. This variant has been consistently associated with low HDL-C concentrations in various reports [13]–[17], and the sole presence of the C230 risk allele explains almost 4% of plasma HDL-C concentration variation, which is higher than all HDL-C variation associated with a single nucleotide polymorphism identified through genome-wide association studies in European and Asian populations [17]. Moreover, although it has been associated with additional cardiovascular risk factors such as increased body mass index (BMI), obesity, metabolic syndrome and type 2 diabetes mellitus (T2DM) [14], [18], it is not known whether it is associated with coronary artery disease (CAD).

The purpose of the present study was to analyze whether the *ABCA1*/R230C variant is associated with premature CAD and subclinical atherosclerosis in a case-control association study: GEA (Genetics of Atherosclerotic Disease). In addition, because obesity is associated with an increased risk of dislipidemia, insulin resistance and hepatic steatosis, we explored whether BMI modulates the effect of the C230 allele on various metabolic traits using a population-based design.

## Materials and Methods

The primary aim of the GEA Study is to investigate genetic factors associated with premature CAD, subclinical atherosclerosis and other coronary risk factors in the Mexican population. All participants provided written informed consent, and the study was approved by the Ethics Committee of the Instituto Nacional de Cardiología “Ignacio Chávez” (INCICH) and the Ethics Committee of the Instituto Nacional de Medicina Genómica (INMEGEN).

### Subjects

All GEA participants are unrelated and of self-reported Mexican-mestizo ancestry (3 generations). To date, a total of 2193 individuals have been included, 949 diagnosed with premature CAD and 1244 apparently healthy controls. Premature CAD was defined as history of myocardial infarction, angioplasty, revascularization surgery or coronary stenosis >50% on angiography, diagnosed before age 55 in men and before age 65 in women. All cases were recruited from the Department of Hemodynamics and Outpatient Clinic at the INCICH in Mexico City, and patients with acute cardiovascular events 3 months prior to the study were excluded. Controls were apparently healthy asymptomatic individuals without family history of premature CAD, recruited from blood bank donors and through brochures posted in Social Services centers. Exclusion criteria for controls included congestive heart failure; liver, renal, thyroid or oncological disease.

All participants answered standardized and validated questionnaires to obtain information on family and medical history, alcohol and tobacco consumption, dietary habits and physical activity [19], [20]. Anthropometric parameters were measured by trained personnel, and included waist circumference and body mass index (BMI) calculated as weight in kilograms divided by height in meters squared. Blood pressure was measured 3 times by sphygmomannometry and the last two measurements were averaged. Obesity was defined as BMI ≥30 kg/m2. Central obesity, hypoalphalipoproteinemia, hypertriglyceridemia and metabolic syndrome were defined using Adult Treatment Panel III (ATP-III) criteria [21]. Hypercholesterolemia was defined as total cholesterol (TC) levels ≥200 mg/dL. Hypertension was defined as systolic blood pressure ≥140 mmHg and/or diastolic blood pressure ≥90 mmHg or the use of oral antihypertensive therapy. T2DM was diagnosed according to World Health Organization criteria [22].

### Biochemical Parameters

Venous blood samples were obtained after a 12-hour fast, and all measurements were performed at the Endocrinology Laboratory of the INCICH using standardized procedures. This laboratory is certified for standardization of tests by the Center for Disease Control in Atlanta, GA. Plasma glucose, total cholesterol (TC), triglyceride (TG), and HDL-C levels were measured with commercially available standardized methods as described by Medina-Urrutia et al. [23]. Low density lipoprotein cholesterol (LDL-C) concentrations were estimated using Friedewald’s formula modified by De Long [24]. Serum insulin concentrations were determined by radioimmunoanalysis (Milipore, RIA Kit, USA) and homeostasis model assessment of insulin resistance (HOMA-IR) was calculated from fasting glucose and insulin measures [25].

### Computed Tomography of the Chest and Abdomen

Computed tomography of the chest and abdomen were performed using a 64-channel multi-detector helical computed tomography system (Somatom Sensation, Siemens) and interpreted by experienced radiologists. Scans were read to assess and quantify the following: 1) Coronary artery calcification (CAC) score using the Agatston method [26]; 2) total abdominal, subcutaneous and visceral adipose tissue areas as described by Kvist [27] in order to calculate visceral to subcutaneous adipose tissue ratio (VAT/SAT); and 3) hepatic to splenic attenuation ratio (LSAR) as described by Longo et al. [28]. Subclinical atherosclerosis (SA) was defined as the presence of coronary calcium (CAC score >0) and hepatic steatosis was defined as LSAR ≤1.0 [29].

### Genetic Analyses

Genomic DNA was extracted and purified from white blood cells using the salting-out procedure [30]. The *ABCA1*/R230C variant (rs9282541) was genotyped using TaqMan assays (ABI Prism 7900HT sequence detection system (Applied Biosystems). Genotyping call exceeded 95% and no discordant genotypes were observed in 184 duplicate samples. Samples previously genotyped by direct sequencing were used as positive controls.

### Statistical Analyses

All calculations were performed using SPSS version 18.0 (SPSS, Chicago, Il) statistical package and R [31]. Means ± SD and frequencies of baseline characteristics were calculated. Chisquare tests were used to compare frequencies and ANOVA and Student′s *t*-test were used to compare means. Lipid traits, HOMA-IR and VAT/SAT ratio were log-transformed due to skewed distribution. ANCOVA was used to determine associations between the R230C variant and metabolic variables, adjusting for age, gender, BMI, and HDL-C levels as indicated using additive and dominant models. Simple linear regression models were built to study the effect of BMI on lipid concentrations, HOMA-IR and VAT/SAT ratio stratified by sex and genotype. To study gene-BMI interactions, we used multivariate linear regression models including main effects and interaction terms under a dominant model. To address multiple testing, Bonferroni’s correction was used considering 12 independent tests and statistical significance was set when p≤0.004. Statistical power to detect association of R230C with premature CAD exceeded 0.80 as estimated with QUANTO software (http://hydra.usc.edu/GxE/). Genotype frequencies did not show deviation from Hardy-Weinberg equilibrium (*P* = 0.14).

## Results

General characteristics of the population are shown in Tables 1 and 2. Because 331 (26.6%) of the apparently healthy individuals recruited as controls showed a positive coronary calcium score, three independent groups were considered for the analysis: controls (CAC score = 0), subclinical atherosclerosis (CAC score >0), and premature CAD.

**Table 1:** Demographic characteristics of the population.

	Controls	SA	Premature CAD	P Value*
	CAC = 0	CAC >0		
	(n = 913)	(n = 331)	(n = 949 )	
Age (years)	51.86±8.92	58.88±8.47	53.51±7.34	**<0.0001**
Gender (% males)	38.1	73.1	80.5	**<0.0001**
Body Mass Index (Kg/m^2^)	28.37±4.47	28.80±4.35	28.86±4.23	0.021
Obesity (%)	31.1	34.4	36.5	0.050
Waist circumference (cm)	93.85±11.70	97.56±11.33	98.30±11.02	**<0.0001**
Central obesity (%)	49.1	43.2	43.0	0.021
Total Abdominal Fat (cm^2^)	448.76±145.88	464.96±153.10	441.22±142.76	NS
Subcutaneous Abdominal Fat (cm^2^)	300.06±113.53	279±112.50	262.51±100.83	**<0.0001**
Visceral Abdominal Fat (cm^2^)	148.76±63.20	185.80±68.27	179.01±72.59	**<0.0001**
Visceral/Subcutaneous adipose tissue ratio	0.553±.0319	0.734±0.318	0.740±0.343	**<0.0001**
Current Smokers (%)	22.3	22.3	12.1	**<0.0001**
Former Smokers (%)	28.1	44.1	63.6	**<0.0001**
Hypertension (%)	21.9	27.6	67.7	**<0.0001**
Hypertensive Medication (%)	14.8	28.6	67.1	**<0.0001**
Diastolic Blood Pressure (mmHg)	71.12±9.09	76.32±10.31	73.08±10.20	**<0.0001**
Systolic Blood Pressure (mmHg)	114.98±16.23	126.41±19.92	119.11±18.84	**<0.0001**
Heart Rate (bpm)	66.13±9.30	66.21±10.13	65.47±11.19	NS

Data are expressed as means ± SD, log-transformed values were used for statistical analysis.

* *P* values were estimated using ANOVA for continuous variables and Pearson’s Chisquare test for categorical values.

CAD: coronary artery disease; SA: subclinical atherosclerosis.

**Table 2:** Comparison of biochemical parameters in individuals with premature coronary artery disease, subclinical atherosclerosis and controls.

	Controls	SA	Premature CAD	P Value*
	CAC = 0	CAC >0		
	(n = 913)	(n = 331)	(n = 949 )	
Total Cholesterol (mg/dL)	192.22±36.64	198.12±38.23	168.70±48.06	**<0.0001**
TC ≥200 mg/dL (%)	37.8	50.2	22.3	**<0.0001**
HDL-C (mg/dL)	48.48±14.16	45.19±12.48	40.17±10.64	**<0.0001**
Hypoα-lipoproteinemia (%)	49.0	46.5	64.7	**<0.0001**
LDL-C (mg/dL)	117.24±31.41	124.48±32.35	97.59±39.55	**<0.0001**
Triglycerides (mg/dL)	168.79±109.49	178.80±102.28	192.59±122.57	**<0.0001**
Hypertriglyceridemia (%)	46.6	54.2	58.9	**<0.0001**
ApoAI (mg/dL)	139.01±37.39	138.75±35.53	120.86±26.82	**<0.0001**
ApoB (mg/dL)	93.42±27.13	98.90±27.80	84.14±31.49	**<0.0001**
Statin and/or Fibrate Treatment (%)	6.9	14.5	95.8	**<0.0001**
Type 2 Diabetes Mellitus (%)	10.4	22.1	35.5	**<0.0001**
Glucose (mg/dL)†	89.72±9.53	92.10±9.54	90.95±9.54	**<0.001**
HOMA-IR†	4.31±2.66	4.61±2.60	5.25±3.33	**<0.0001**
Hepatic Steatosis (%)	33.2	38.5	34.0	NS
Alanine Transaminase (IU/L)	29.39±20.09	27.44±17.19	29.23±17.59	NS
Aspartate Transaminase IU/L)	27.64±11.98	28.11±13.21	28.06±10.99	NS
Alkaline Phosphatase (IU/L)	83.91±25.09	81.69±30.70	80.07±25.73	**<0.001**
Gamma-glutamyl transpeptidase (IU/L)	36.72±39.23	39.084±34.41	44.67±42.68	**<0.0001**

Data are expressed as means ± SD, log-transformed values were used for statistical analysis.

* *P* values were estimated using ANOVA for continuous variables and Pearson’s Chisquare test for categorical values.

† Individuals with diagnosis of T2D were excluded from the analysis.

CAD: coronary artery disease; SA: subclinical atherosclerosis.

### Association of ABCA1/R230C with Premature CAD

The C230 risk allele frequency was similar in controls and individuals with subclinical atherosclerosis (.106 and.093 respectively), but lower in the premature CAD group (0.072). C230 was significantly associated with a lower risk of premature CAD as compared to controls under both dominant and additive models adjusting for age, gender and BMI (OR = 0.669, 95% CI:0.508–0.882, *P_dom_* = 0.004 and OR = 0.655, 95% CI:0.509–0.843, *P_add_* = 0.001). In addition, individuals with subclinical atherosclerosis showed a marginally significant decreased risk of premature CAD adjusting for age, gender and BMI (OR = 0.690, 95% CI: 0.486–0.979, *P_dom_* = 0.038 and OR = 0.719, 95% CI: 0.520–0.994, *P_add_* = 0.046). When multiple regression models included HDL-C levels as a covariate, the associations were also significant (*P_dom_* = 1.210×10^−4^ and *P_add_* = 1.499×10^−5^ as compared to controls; *P_dom_* = 0.007 and *P_add_* = 0.008 as compared to individuals with subclinical atherosclerosis) (Table 3).

**Table 3:** Association of the R230C/*ABCA1* variant with premature coronary artery disease and subclinical artherosclerosis.

	RRGENOTYPE FREQUENCY	RC GENOTYPE FREQUENCY	CC GENOTYPE FREQUENCY	MAF	MODEL	OR (95% CI)	P value
CONTROLS (n = 913)	.805	.179	.017	.106			
SA(n = 331)	.820	.174	.006	.093	Dominant	0.993 (.981–1.005)†	NS
					Additive	0.880 (.630–1.229)†	NS
PREMATURE CAD(n = 949)	.860	.135	.005	.072	Dominant	0.576 (.434–.763)†	**1.210×10^−4^**
					Additive	0.566 (0.437–.732)†	**1.499×10^−5^**
					Dominant	0.614 (.430–.873)‡	**0.007**
					Additive	0.643 (.463–.893)‡	**0.008**

Associations were tested using logistic regression adjusting for age, gender, BMI and HDL-C levels.

SA: subclinical atherosclerosis; CAD: coronary artery disease; MAF: minor allele frequency.

† Compared to controls.

‡ Compared to individuals with subclinical atherosclerosis.

### Association of ABCA1/R230C with Metabolic Traits

The effect of the C230 risk allele on various metabolic parameters was explored in all individuals recruited initially as controls regardless of their CAC score. As previously reported, C230 was significantly associated with lower HDL-C levels (*P_dom_ = *9.819×10^−9^; *P_add_* = 1.242×10^−9^) and showed a marginal association with lower total cholesterol levels (*P_dom_* = 0.027; *P_add_* = 0.013), but was not independently associated with BMI or VAT/SAT ratio (Table 4). Moreover, C230 was associated with hypoalphalipoproteinemia (*P_dom_ = *1.232×10^−7^; *P_add_* = 1.069×10^.8^), metabolic syndrome (*P_dom_ = *0.001; *P_add_* = 0.001), and a marginal decreased risk of hypercholesterolemia (*P_dom_* = 0.050; *P_add_* = 0.029), but was not associated with obesity, T2DM or hepatic steatosis in the entire sample (Table 5) or stratified by gender.

**Table 4:** Association of the R230C/*ABCA1* variant with quantitative metabolic parameters.

	RR GENOTYPE	RC+CC GENOTYPES	MODEL	β	95% CI Inferior	95% CI Superior	P value
BMI (Kg/m^2^)	28.60±4.45	27.99±4.36	Dominant	−0.009	−0.019	0.001	NS
			Additive	−0.008	−0.016	0.001	NS
HDL-C (mg/dL)	48.51±13.76	44.04±13.15	Dominant	−0.046	−0.062	−0.030	**9.819×10^−9^**
			Additive	−0.043	−0.057	−0.029	**1.242×10^−9^**
TC (mg/dL)	194.91±37.62	188.51±34.32	Dominant	−0.013	−0.025	−0.002	**0.027**
			Additive	−0.013	−0.024	−0.003	**0.013**
TG (mg/dL)	169.20±107.17	176.34±106.66	Dominant	0.021	−0.007	0.049	NS
			Additive	0.016	−0.009	0.041	NS
HOMA-IR	5.17±7.92	4.98±3.43	Dominant	−0.015	−0.053	0.023	NS
			Additive	−0.013	−0.047	0.022	NS
VAT/SAT ratio	0.599±0.334	0.611±0.318	Dominant	0.009	−0.030	0.048	NS
			Additive	0.008	−0.028	0.043	NS

Data are expressed as means ± standard deviation. Linear models were used adjusting for age, gender and BMI when appropriate based on log-transformed values.

TC: total cholesterol; TG: triglycerides; VAT/SAT ratio: visceral to subcutaneous adipose tissue ratio.

**Table 5:** Associations of the R230C/*ABCA1* variant with metabolic risk factors for coronary artery disease.

	MAF Controls	MAF Cases	MODEL	OR (95% CI)	P-Value
Obesity (n = 387)	0.108	0.093	Dominant	0.762 (0.556–1.044)	NS
			Additive	1.212 (0.914–1.608)	NS
Hypoαlipoproteinemia (n = 549)	0.073	0.142	Dominant	2.221 (1.652–2.985)	**1.232×10^−7^**
			Additive	2.220 (1.689–2.918)	**1.069×10^−8^**
Hypercholesterolemia (n = 505)	0.115	0.086	Dominant	0.743 (0.553–1.000)	**0.050**
			Additive	0.740 (0.565–0.969)	**0.029**
Hypertriglyceridemia (n = 593)	0.096	0.111	Dominant	1.276 (0.953–1.707)	NS
			Additive	0.920 (0.731–1.066)	NS
Metabolic Syndrome(n = 407)	0.094	0.123	Dominant	1.725 (1.244–2.392)	**0.001**
			Additive	1.630 (1.216–2.184)	**0.001**
Hepatic Steatosis (n = 416)	0.106	0.097	Dominant	0.939 (0.683–1.292)	NS
			Additive	1.035 (0.778–1.379)	NS
Type 2 Diabetes Mellitus (n = 167)	0.102	0.103	Dominant	1.032 (0.909–1.788)	NS
			Additive	0.955 (0.649–1.405)	NS

All associations were tested using logistic regression adjusting for age, gender and BMI when appropriate. (n) represents the number of cases with each trait.

MAF: minor allele frequency.

### Correlation between BMI and Metabolic Traits According to Genotype

#### BMI and VAT/SAT ratio

**Figure 1:**
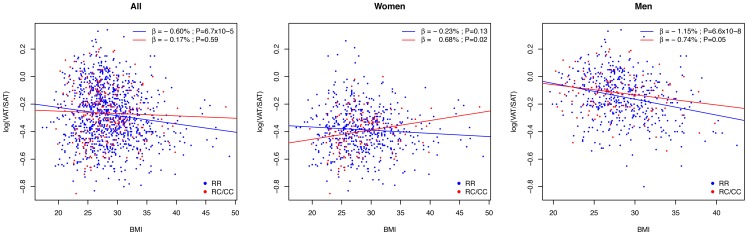
Correlation between Abdominal Fat Distribution and Body Mass Index (BMI) According to Genotype. Lines represent simple linear regressions: blue lines represent RR genotypes and red lines represent C230 risk allele carriers (RC/CC genotypes). Overall, body mass index (BMI) was negatively correlated with visceral to subcutaneous adipose tissue ratio (VAT/SAT) in individuals with RR genotypes, but not in individuals with RC or CC genotypes. On gender stratification, a positive and significant correlation of BMI and VAT/SAT was observed only in women bearing the C230 allele. BMI-VAT/SAT correlations were negative in men with and without the C230 allele.

**Figure 2:**
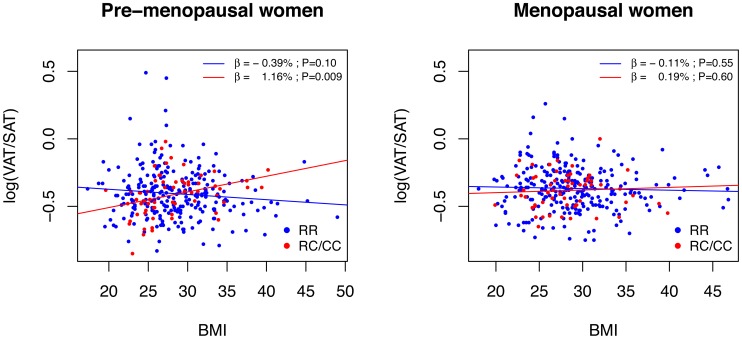
The Interaction of R230C and BMI Affects the Distribution of Abdominal Fat in Premenopausal Women. Lines represent simple linear regressions, blue lines represent RR genotypes and red lines represent C230 risk allele carriers (RC/CC genotypes). Premenopausal women with RR genotypes show a non-significant negative BMI-VAT/SAT correlation; however visceral fat correlated positively and significantly with BMI only in premenopausal women with RC and CC genotypes. BMI showed no correlation with abdominal fat distribution in menopausal women.

**Figure 3:**
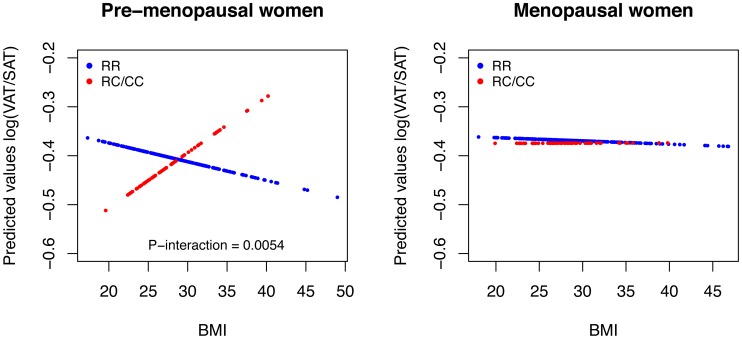
Predicted VAT/SAT Ratio Values According to BMI in Premenopausal and Menopausal Women. Predicted visceral to subcutaneous adipose tissue ratio (VAT/SAT) values were calculated from regression models containing the *ABCA1*/R230C variant, BMI and the interaction term, adjusted for age. Blue lines represent RR genotypes and red lines represent C230 risk allele carriers (RC/CC genotypes). The interaction between the polymorphism and BMI was significant only in premenopausal women (*P* = 0.005).

Overall, BMI showed a statistically significant negative correlation with VAT/SAT ratio in individuals with RR genotypes (β = −0.60%; *P* = 6.7×10^−5^), but not in individuals with RC or CC genotypes (β = −0.17%; *P* = 0.59) (Figure 1). On gender stratification, a positive and significant correlation of BMI and VAT/SAT ratio was observed only in women bearing the C230 allele (β = 0.68%; *P* = 0.02), particularly in premenopausal (β = 1.16%; *P* = 0.009), but not in menopausal women (β = −0.19%; *P* = .60) (Figure 2). Predicted values were calculated from regression models containing the *ABCA1*/R230C variant, BMI and the interaction term, adjusted for age (Figure 3). The interaction between the polymorphism and BMI was significant only in premenopausal women (*P* = 0.005). No significant BMI-R230C interactions were observed for LSAR (data not shown).

#### BMI and HOMA-IR

BMI and HOMA-IR showed positive and significant correlations in both genders, and no significant differences according to *ABCA1*/R230C genotype were observed in the entire sample or stratifying by gender (Figure S1, A and B). In premenopausal women bearing R230C genotypes, this effect showed a modest increase (β = 3.74%; *P* = 4.9×10^−6^) as compared to R230R homozygous premenopausal women (β = 2.55%; *P* = 1.2×10^−13^) (Figure S1, C and D), although the interaction did not reach statistical significance (*P* = 0.121). Predicted values were calculated from regression models containing the *ABCA1/*R230C variant, BMI and the interaction term in premenopausal women (Figure S1, E and F).

#### BMI and lipid traits

BMI showed a positive correlation with TG levels in both men and women (Figure S2, A and B). Men showed no differences according to genotype (β = 1.14%; *P* = 2×10^−5^ and β = 1.25%; *P* = 0.004 for RR and RC/CC genotypes respectively). However, the effect of BMI on TG levels was greater in women carrying the risk allele (β = 1.61%; *P* = 1.3×10^−4^) than in women with RR genotypes (β = 0.72%; *P* = 6.0×10^−6^). Interestingly, the effect of BMI in women carrying the C230 allele did not differ according to menopausal status (Figure S2, C and D). Predicted TG values were calculated from regression models containing the *ABCA1*/R230C variant, BMI and the interaction term, adjusted for age (Figure S2, E), and the interaction between BMI and the R230C variant was significant only in women (*P* = 0.036). BMI did not modulate the effect of the C230 allele on HDL-C or TC levels in any gender (data not shown).

## Discussion

The GEA study is the first study in the population of Mexico City specifically designed to seek genetic factors associated with premature coronary artery disease and subclinical atherosclerosis. Because the control and subclinical atherosclerosis groups were recruited from volunteers of Mexico City, and because of the fine clinical and metabolic characterization, this cohort is also useful to study metabolic cardiovascular risk factors in a population-based design.

### Association with Decreased Risk of Premature CAD

Many studies have sought to associate *ABCA1* genetic variants with the risk of HDL-C levels, atherosclerosis and coronary artery disease [5]–[12], and there is evidence that *ABCA1* variation predicts ischemic heart disease in the general population [12]. However, despite the strong and consistent inverse relation between plasma HDL-C levels and cardiovascular risk, it has recently been questioned whether this is a causal association [2], [11]. Firstly, there is large discrepancy between the virtual absence of plasma HDL-cholesterol in Tangier patients and the lack of the expected large increase in the risk of cardiovascular disease predicted from epidemiological studies [5], [32]. Secondly, there is discordance between genetic variants affecting HDL-C levels *per se* and cardiovascular risk. In this regard, heterozygosity for loss of function *ABCA1* mutations were associated with lower plasma HDL-cholesterol levels, but not with an increased risk of ischemic heart disease after adjusting for known cardiovascular risk factors [10]; *ABCA1* variants V772M and V825I were both associated with increased HDL-C levels and increased IHD risk [12], and the *ABCA1* promoter variant rs2422498 was associated with a decreased risk of 10-year vascular death in CAD patients with no apparent effect on HDL-C levels [33].

The results of the present case-control association study is another example of this discrepancy, as the *ABCA1*/R230C variant was significantly associated with both decreased HDL-C levels and a decreased risk of premature CAD. Interestingly, the association with premature CAD was significant with and without adjusting for HDL-C levels as a covariate, suggesting that the effects of *ABCA1*/R230C on HDL-C levels and the risk of premature CAD are independent. Moreover, the C230 allele was not associated with subclinical atherosclerosis, and individuals with subclinical atherosclerosis showed a marginally significant decreased risk of premature CAD adjusting for age, gender and BMI. Because *ABCA1* has many functions in distinct cell types [34], these discrepancies may be due to a pleiotropic effect of *ABCA1* variants in other cell types involved in the pathophysiology of atherosclerosis and CAD. It is known that *ABCA1* is involved in inflammation [35]–[37], and various alterations have been reported in *ABCA1*-deficient platelets and Tangier patients including mild thrombocytopenia and bleeding tendencies [38], [39]. To date it is not known whether C230 or other *ABCA1* variants affect these or other functions involved in atherosclerosis or CAD pathogenesis. Further research on the functional consequences of *ABCA1* variants in platelets, endothelial function, inflammation and other tissues may offer explanations for this paradox, and as to why the effects of these variants on HDL-C levels and cardiovascular risk are independent.

### Associations with other Metabolic Parameters

To date, all reports assessing the effect of the *ABCA1*/R230C variant on HDL-C concentrations including the present study have consistently shown highly significant associations with decreased HDL-C levels [13]–[17]. However, associations with other metabolic parameters such as obesity, T2DM and TG levels have been inconsistent. Because obesity is associated with many metabolic risk factors for cardiovascular disease, we sought possible explanations for such inconsistencies exploring whether BMI modulates the effect of the R230C variant on several metabolic traits.

We found no evidence of BMI modulating the effect of *ABCA1*/R230C on any of the metabolic parameters explored in men. However, some BMI-R230C interactions were observed in women: BMI modulated the effect of this allele on VAT/SAT ratio and HOMA-IR in pre-menopausal women and on TG levels in women regardless of their menopausal status. The VAT/SAT ratio is a metric of propensity to store visceral as compared to subcutaneous fat with known gender differences and associated with cardiometabolic risk [40], [41]. Accordingly, the correlation between BMI and HOMA-IR was higher in pre-menopausal women carrying the C230 allele, although the interaction did not reach statistical significance. The BMI-R230C interactions observed only in pre-menopausal women suggest that these effects may be estrogen-related. Several lines of evidence are consistent with this finding: *ABCA1*-diet interactions affecting HDL-C levels have been reported in pre-menopausal women [17], improved lipid profiles have been observed in response to estrogen [42]–[43], and estrogen increased *ABCA1* expression in different tissues in both mice and humans [44]–[46]. The increased risk of these metabolic parameters in pre-menopausal women is again in discrepancy with their risk of CAD, and whether this has to do with other systemic effects of estrogen, and/or with pleiotropic effects of the R230C variant in platelets, endothelium, inflammatory or other cell types remains to be elucidated.

The R230C allele was associated with T2DM in a case-control association study in the Mexican population and replicated in an independent sample [18]. However other population-based studies have not found evidence of an increased risk of T2DM for C230 allele carriers [16], [17], including the present study. This may be due to differences in study design; however it is likely that diet, physical exercise, gender and other environmental factors may modify the effect of this functional allele on glucose and lipid metabolism, affecting the risk of T2DM. Although the BMI-C230 allele interaction did not reach significance for HOMA-IR, further studies with larger sample sizes may elucidate whether there is such an interaction and if it might affect T2DM risk. Further extensive research on gene-diet, gene-physical activity and other gene-environment interactions is required to understand how *ABCA1* variants affect the risk of T2DM, obesity and other metabolic traits.

### Conclusion

This is the first study assessing the effect of the R230C/*ABCA1* variant in premature coronary artery disease. This variant was associated with both decreased HDL-C levels and a lower risk of premature CAD, and gender-specific BMI-R230C variant interactions were observed for different metabolic traits. These findings underscore the need to further analyze interactions of this functional and frequent variant with diet, exercise and other environmental factors.

## Supporting Information

Figure S1 **Correlation between HOMA-IR and BMI according to genotype.** Lines represent simple linear regressions. Blue lines represent RR genotypes and red lines represent C230 risk allele carriers (RC/CC genotypes). Body mass index (BMI) showed a significant and positive correlation regardless of genotype in all women (A), men (B), premenopausal women (C) and menopausal women (D). This effect showed a modest increase in premenopausal women bearing the C230 allele, although the interaction did not reach statistical significance (*P* = 0.12). Predicted HOMA-IR values were calculated from regression models containing the *ABCA1*/R230C variant, BMI and the interaction term in premenopausal (E) and menopausal women (F).
(TIF)

Figure S2 **The interaction of R230C and BMI affects triglyceride levels in women.** Lines represent simple linear regressions. Blue lines represent RR genotypes and red lines represent C230 risk allele carriers (RC/CC genotypes). Body mass index (BMI) showed a significant and positive correlation with triglyceride levels regardless of genotype in all women (A), men (B), premenopausal women (C) and menopausal women (D). However, this effect increased in women bearing the C230 allele regardless of menopausal status. Predicted triglyceride values (E) were calculated from regression models containing the *ABCA1*/R230C variant, BMI and the interaction term in women, and the interaction was statistically significant (*P* = 0.036).
(TIF)
