# Supplementary figures and images for: The ABCA1 Gene R230C Variant Is Associated with Decreased Risk of Premature Coronary Artery Disease: The Genetics of Atherosclerotic Disease (GEA) Study
Source: PLoS One. 2012 Nov 9;7(11):e49285. doi: 10.1371/journal.pone.0049285 (PMC3494680; doi:10.1371/journal.pone.0049285)

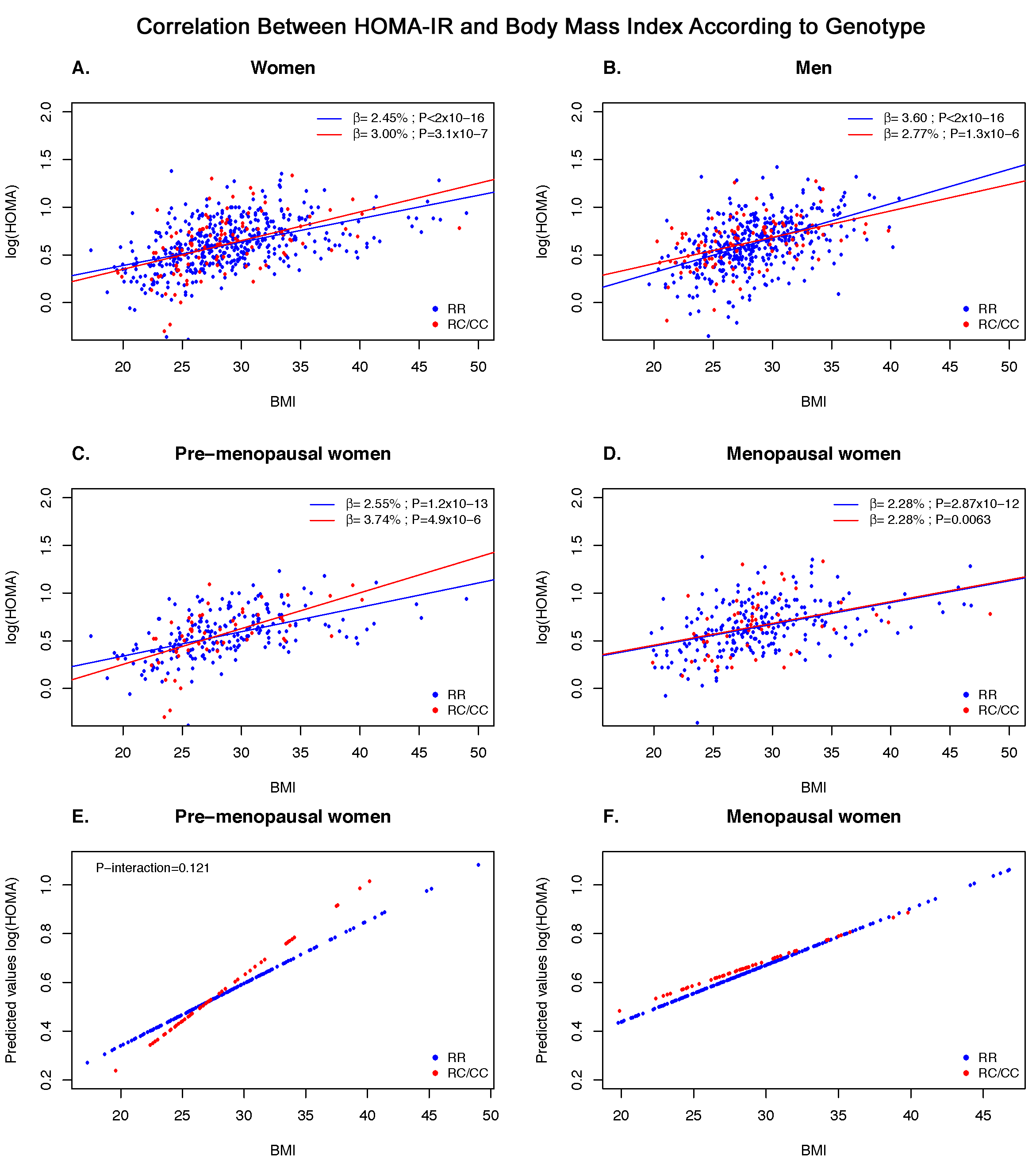

Supplement: Figure S1 — Correlation between HOMA-IR and BMI according to genotype. Lines represent simple linear regressions. Blue lines represent RR genotypes and red lines represent C230 risk allele carriers (RC/CC genotypes). Body mass index (BMI) showed a significant and positive correlation regardless of genotype in all women (A), men (B), premenopausal women (C) and menopausal women (D). This effect showed a modest increase in premenopausal women bearing the C230 allele, although the interaction did not reach statistical significance (P = 0.12). Predicted HOMA-IR values were calculated from regression models containing the ABCA1/R230C variant, BMI and the interaction term in premenopausal (E) and menopausal women (F). (TIF) [file pone.0049285.s001.tif]

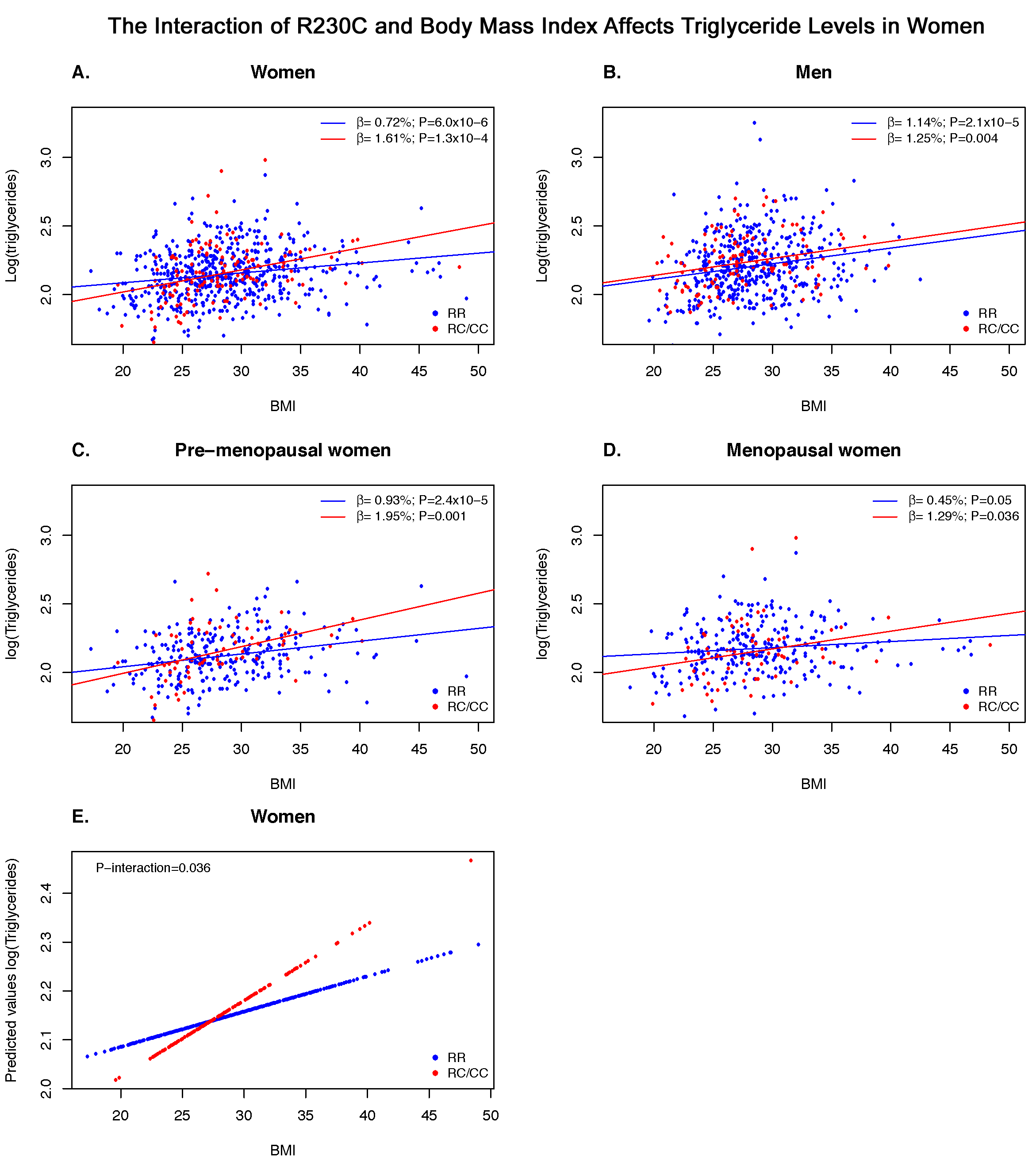

Supplement: Figure S2 — The interaction of R230C and BMI affects triglyceride levels in women. Lines represent simple linear regressions. Blue lines represent RR genotypes and red lines represent C230 risk allele carriers (RC/CC genotypes). Body mass index (BMI) showed a significant and positive correlation with triglyceride levels regardless of genotype in all women (A), men (B), premenopausal women (C) and menopausal women (D). However, this effect increased in women bearing the C230 allele regardless of menopausal status. Predicted triglyceride values (E) were calculated from regression models containing the ABCA1/R230C variant, BMI and the interaction term in women, and the interaction was statistically significant (P = 0.036). (TIF) [file pone.0049285.s002.tif]
